# Acurácia Diagnóstica do Teste Ergométrico para Detectar Via Acessória de Alto Risco em WPW: Uma Revisão Sistemática e Metanálise

**DOI:** 10.36660/abc.20240663

**Published:** 2025-04-17

**Authors:** José Nunes de Alencar, Fabio Mahamad Rassi, Raquel Pereira Rios, Matheus Kiszka Scheffer, Guilherme Dagostin de Carvalho

**Affiliations:** 1 Instituto Dante Pazzanese de Cardiologia São Paulo SP Brasil Instituto Dante Pazzanese de Cardiologia, São Paulo, SP – Brasil; 2 Escola Paulista de Medicina Universidade Federal de São Paulo São Paulo SP Brasil Escola Paulista de Medicina da Universidade Federal de São Paulo, São Paulo, SP – Brasil

**Keywords:** Síndrome de Wolff-Parkinson-White, Teste de Esforço, Revisão Sistemática, Metanálise

## Abstract

**Fundamento:**

A síndrome de Wolff-Parkinson-White (WPW) é caracterizada por pré-excitação ventricular, que pode levar a eventos arrítmicos graves, como taquicardia supraventricular e fibrilação atrial pré-excitada. O valor diagnóstico de testes ergométricos não invasivos para detectar vias acessórias de alto risco permanece inconsistente na literatura.

**Objetivos:**

Avaliar a precisão diagnóstica de testes ergométricos não invasivos em comparação com os estudos eletrofisiológicos invasivos (EEF) para identificar vias acessórias de alto risco na síndrome de WPW.

**Métodos:**

De acordo com as diretrizes do PRISMA-DTA, foi realizada uma busca abrangente nas bases de dados PubMed, Scopus e Web of Science. Estudos elegíveis avaliaram a sensibilidade, especificidade e razões de verossimilhança de testes ergométricos não invasivos em pacientes com WPW, utilizando EEF como padrão de referência. Para a metanálise, foi aplicado um modelo bivariado de efeitos aleatórios.

**Resultados:**

Seis estudos, totalizando 765 pacientes, atenderam aos critérios de inclusão. A sensibilidade combinada foi de 92,7% (IC de 95%: 88,0% – 94,0%), e a especificidade combinada foi de 28,1% (IC de 95%: 23% – 35,1%). A razão de verossimilhança negativa (LR-) de 0,260 (IC de 95%: 0,174 – 0,387) indicou que a presença de uma via acessória de alto risco é aproximadamente quatro vezes menos provável após um resultado de teste negativo. A análise de sensibilidade, restrita a pacientes pediátricos, demonstrou resultados consistentes.

**Conclusão:**

Testes ergométricos não invasivos demonstram uma utilidade diagnóstica razoável para descartar vias de alto risco na síndrome de WPW. No entanto, é necessária cautela ao utilizar esses testes como critérios independentes para estratificação de risco.

## Introdução

A pré-excitação ventricular, uma condição que afeta cerca de 0,1% dos neonatos,^1^ pode manifestar-se clinicamente ao longo da vida com sintomas que variam de palpitações e síncope até desfechos mais graves, como a morte cardíaca súbita. Isso ocorre, em grande parte, devido à sua associação com taquicardia supraventricular e fibrilação atrial. Pacientes diagnosticados com síndrome de Wolff-Parkinson-White (WPW) apresentam uma taxa de mortalidade consideravelmente mais alta, com incidência de morte súbita estimada em aproximadamente 0,15% ao ano, podendo aumentar para 3–4% ao longo da vida.^2^

As características clínicas e eletrofisiológicas associadas ao aumento do risco de morte cardíaca súbita na síndrome de WPW dependem da capacidade de condução atrioventricular rápida da via acessória. Os principais indicadores de risco elevado incluem um intervalo RR pré-excitado mais curto (SPERRI) < 250 ms ou um período refratário efetivo anterógrado da via acessória (APERP) notavelmente reduzido, variando entre 220–270 ms.^3-7^ Além disso, a normalização abrupta e completa do intervalo PR, acompanhada do desaparecimento da onda delta durante o teste ergométrico, tem sido tradicionalmente reconhecida como um marcador de baixo risco.^8,9^ A avaliação não invasiva das propriedades condutoras da via acessória pode ser considerada (Classe IIb) em indivíduos com pré-excitação assintomática, conforme as diretrizes da ESC.^7^

Esta revisão sistemática e metanálise segue as diretrizes PRISMA-DTA^10^ e tem como objetivo combinar e analisar as evidências entre estudos para avaliar a sensibilidade, especificidade, razões de verossimilhança e razões de probabilidade diagnóstica de testes ergométricos nesse contexto.

## Métodos

O protocolo desta revisão sistemática e metanálise da acurácia diagnóstica (DTA) foi registrado no Registro Prospectivo Internacional de Revisões Sistemáticas (PROSPERO). O número de registro para acessar o protocolo é CRD42024526932.

Realizamos uma pesquisa completa nas bases de dados PubMed, Scopus e Web of Science, com a última busca realizada em 20/03/24. A estratégia de busca foi projetada para abranger termos relacionados à síndrome de WPW, testes ergométricos não invasivos e resultados diagnósticos. Para o PubMed, foram incluídos termos como “Síndrome de Wolff-Parkinson-White”, “pré-excitação”, “teste ergométrico”, “APERP”, “SPERRI” e outros termos relacionados. Estratégias semelhantes foram adaptadas para Scopus e Web of Science, considerando a sintaxe e as capacidades de busca de cada base de dados.

Os estudos elegíveis para inclusão foram aqueles que avaliaram a acurácia diagnóstica de testes ergométricos não invasivos na detecção de vias acessórias de alto risco em pacientes com síndrome de Wolff-Parkinson-White (WPW), utilizando estudos eletrofisiológicos invasivos (EEF) como padrão de referência. Foram considerados participantes de qualquer faixa etária com diagnóstico de síndrome de WPW, submetidos a testes ergométricos não invasivos e EEF invasivos. Os principais resultados analisados foram a sensibilidade, especificidade, razão de verossimilhança positiva e razão de verossimilhança negativa dos testes ergométricos na previsão do risco de arritmia. Foram incluídos estudos observacionais, análises retrospectivas e estudos de coorte prospectivos, publicados em qualquer idioma, desde o início até o presente momento. Os critérios de exclusão foram revisões, relatos de casos e estudos sem medidas claras de resultados diagnósticos ou uma comparação direta entre o teste índice e o padrão de referência.

A fase de triagem de títulos da nossa revisão sistemática foi conduzida por dois pesquisadores independentes (RR e FR) usando a plataforma HubMeta.^11^ Quaisquer discrepâncias identificadas durante a triagem inicial foram resolvidas por um terceiro pesquisador independente (MS). A triagem do texto completo foi então realizada por outra dupla de pesquisadores independentes (JA e GD). Nos casos em que surgiram divergências, estas foram resolvidas por meio de discussão entre os autores para chegar a um consenso.

Durante a fase de extração de dados de nossa revisão sistemática, encontramos uma inconsistência recorrente na literatura em relação às definições do que constitui um teste positivo e como o status de “doença” é determinado, o que afeta a classificação de verdadeiros e falsos positivos, bem como verdadeiros e falsos negativos.^12^ Comumente, os estudos consideram um teste positivo quando há uma perda repentina de pré-excitação ventricular no ECG durante o exercício, rotulando os indivíduos como de “baixo risco”, caso identificados por um APERP/SPERRI > 250 ms. Em nossa abordagem, classificamos indivíduos confirmados como de baixo risco (APERP/SPERRI > 250 ms) que perdem a pré-excitação como “verdadeiros negativos”, o que significa que estão “verdadeiramente ausentes de risco”. Consequentemente, definimos um teste positivo como aquele em que a pré-excitação não é perdida, e um indivíduo “verdadeiramente doente” (“verdadeiro positivo”) é aquele de alto risco, identificado por um APERP/SPERRI ≤ 250 ms.

Esse ajuste significa que o que medimos como sensibilidade em nosso estudo corresponde ao que os autores originais poderiam ter relatado como especificidade e, da mesma forma, o valor preditivo positivo (VPP) e o valor preditivo negativo (VPN) foram invertidos. Esta decisão foi desafiadora, mas fundamental, pois acreditamos que produzirá resultados mais robustos e facilitará um melhor entendimento entre a comunidade médica em relação à estratificação de risco na síndrome de WPW.

Para avaliar o risco de viés e as preocupações com a aplicabilidade em cada estudo, utilizamos a ferramenta QUADAS-2.^13^ Esta avaliação completa abordou vários aspectos, incluindo a seleção dos pacientes, o teste índice, o padrão de referência e o fluxo/tempo. Além disso, empregamos a ferramenta visual Robvis para exibir avaliações de risco de viés nos estudos.^14^

### Análise estatística

Os dados dos estudos foram organizados em uma planilha do Excel que captura métricas essenciais, como verdadeiros positivos, falsos positivos, verdadeiros negativos e falsos negativos. Para garantir a acurácia e integridade das informações extraídas, foram feitos esforços para contatar os autores dos estudos a fim de obter esclarecimentos ou dados adicionais. Em seguida, um modelo de efeitos aleatórios bivariados foi utilizado para reunir estimativas de sensibilidade e especificidade entre os estudos.^15,16^ Essa abordagem leva em consideração a potencial heterogeneidade e a correlação entre sensibilidade e especificidade dentro de cada estudo. O modelo também calculou achados relacionados, incluindo razões de verossimilhança e a Razão de Probabilidades Diagnósticas (DOR). A razão de verossimilhança positiva (LR+) quantifica o quanto a probabilidade de doença aumenta com um resultado de teste positivo. Em contrapartida, a razão de verossimilhança negativa (LR-) reflete o quanto a probabilidade de doença diminui com um resultado de teste negativo. Essas métricas são consideradas mais aplicáveis à prática clínica do que a sensibilidade e a especificidade, pois incorporam uma estrutura de raciocínio probabilístico.^17^ A DOR pode ser interpretada como a razão entre as probabilidades de doença em testes positivos em relação às probabilidades de doença em testes negativos, fornecendo uma medida única da eficácia do teste.^18^

As análises foram facilitadas pelo software MetaDTA (versão 2.0.5),^19,20^ que é projetado especificamente para metanálises de acurácia diagnóstica. Os gráficos de floresta foram usados para representar visualmente as distribuições de sensibilidade e especificidade entre os estudos e suas estimativas agrupadas.

Para quantificar a heterogeneidade estatística, utilizamos a estatística Bayesiana^21,22^ e a área da elipse de predição de 95%.^23^

## Resultados

**Figura 1:**
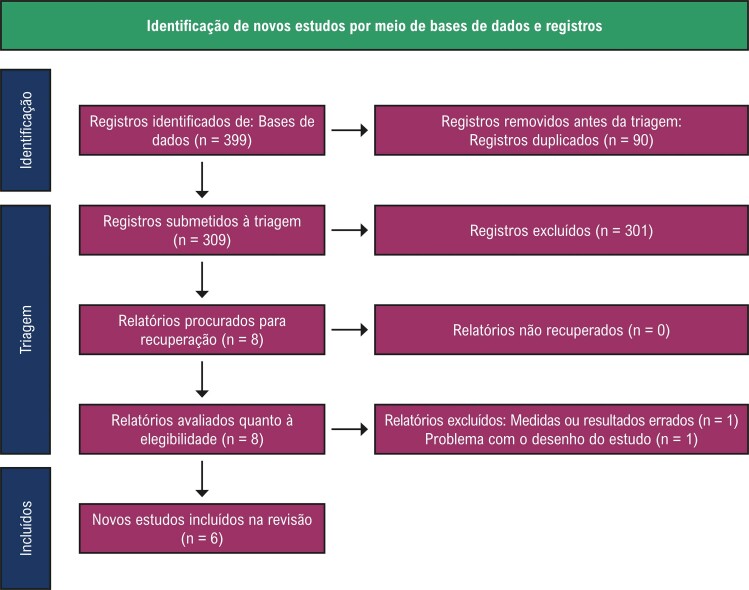
– Fluxograma PRISMA de seleção de estudos para revisão sistemática e metanálise.

Nossa revisão sistemática e metanálise incluiu seis estudos,^24-29^ abrangendo um total de 765 pacientes (Figura 1). Os detalhes desses estudos estão resumidos na Tabela 1.

**Tabela 1:** – Dados dos estudos

Estudo	População	Tamanho da amostra	Teste índice	Número de pacientes testados	Teste de referência
Dalili et al.,^26^ 2014	Pacientes pediátricos	37	Perda de pré-excitação no teste ergométrico	27	SPERRI e APERP < 250 ms
Spar et al.,^24^ 2012	Idade < 21 anos	76	Perda repentina de pré-excitação no teste ergométrico	76	APERP < 270 ms
Jemtrén et al.,^29^ 2024	Idade média de 39 anos, pacientes sintomáticos e assintomáticos	164	Perda repentina de pré-excitação no teste ergométrico	164	APERP ou SPERRI ≤ 250 ms
Wackel et al.,^25^ 2012	Pacientes pediátricos	135	Baixo risco em qualquer teste não invasivo	76	APERP ou SPERRI ≤ 250 ms
Ergul et al.,^27^ 2015	Pacientes pediátricos	40	Perda repentina de pré-excitação no teste ergométrico	40	SPERRI e APERP < 250 ms
Escudero et al.,^28^ 2020	Idade < 21 anos	1589	Perda repentina de pré-excitação no teste ergométrico	382	SPERRI e APERP < 250 ms

Dados iniciais de estudos individuais.

**Figura 2:**
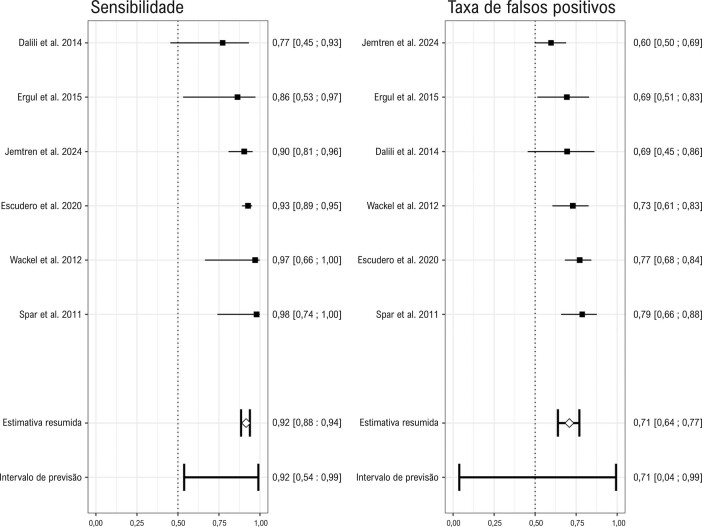
– Gráficos de floresta representando a sensibilidade e a especificidade 1 (taxas de falsos positivos) de cada estudo incluído na detecção de perda repentina de pré-excitação durante o teste ergométrico como um marcador para vias acessórias de baixo risco. Cada ponto estima a sensibilidade e a especificidade 1 de um estudo individual, acompanhado por intervalos de confiança (ICs). A linha inferior descreve o intervalo de previsão, refletindo a faixa esperada de sensibilidades se o teste fosse aplicado em diferentes configurações. As estimativas combinadas são discutidas no texto principal.

Em relação ao APERP ou SPERRI, a sensibilidade combinada, que mede a capacidade do teste de detectar verdadeiros positivos (aqueles que não perdem a pré-excitação ventricular durante o teste ergométrico) em indivíduos de alto risco (aqueles com APERP/SPERRI ≤ 250 ms), foi de 92,7%. A especificidade combinada, indicando a capacidade do teste de identificar verdadeiros negativos (aqueles que perdem a pré-excitação ventricular) entre indivíduos de baixo risco, foi de 28,1%. A Figura 2 resume as estimativas pontuais e os intervalos de confiança de 95%. A razão de verossimilhança positiva (LR+) foi de 1,29 (IC de 95%: 1,179 – 1,411), e a razão de verossimilhança negativa (LR-) foi de 0,260 (IC de 95%: 0,174 – 0,387). A DOR foi de 4,962 (IC de 95%: 3,122 – 7,885).

**Figura 3:**
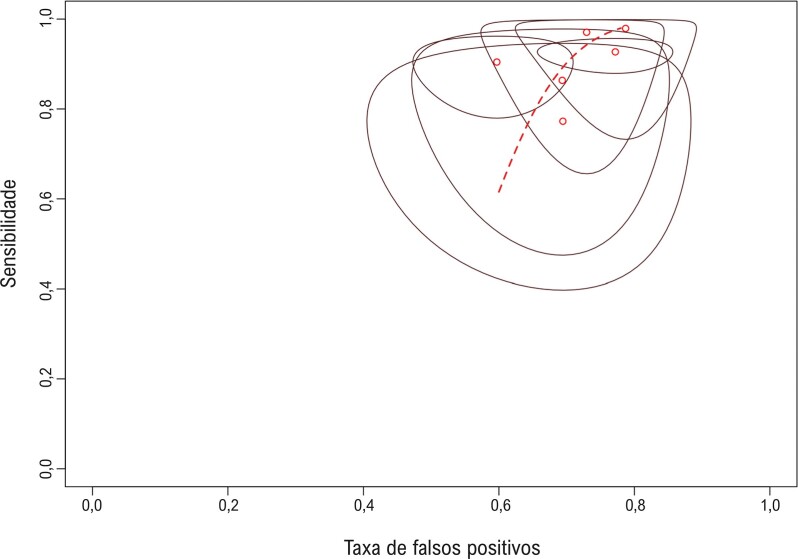
– Curva Característica de Operação do Receptor Sumarizada (SROC) exibindo a compensação entre sensibilidade e taxa de falsos positivos para prever vias acessórias de alto risco. As curvas SROC resumem a acurácia geral do diagnóstico.

Em termos de análise de heterogeneidade, observamos um índice Bayesiano I^2^ de 29% para sensibilidade e 77% para especificidade. A área da elipse na curva Resumo de Característica Operacional do Receptor (SROC) foi de 0,046, indicando um baixo nível de heterogeneidade (Figura 3).

Na análise de sensibilidade, foi pertinente excluir o estudo de Jemtrén et al.,^29^ que incluiu exclusivamente adultos com mais de 21 anos. Esta análise de sensibilidade teve como objetivo avaliar a acurácia do estudo na população pediátrica, e os resultados foram os seguintes: A sensibilidade combinada foi de 92,3% (IC de 95%: 88,8% – 94,8%), e a especificidade combinada foi de 28,4% (IC de 95%: 21,3% – 36,8%). A razão de verossimilhança positiva (LR+) foi de 1,29 (IC de 95%: 1,161 – 1,433), e a razão de verossimilhança negativa (LR-) foi de 0,270 (IC de 95%: (0,179 – 0,408).

Em relação ao uso do SPERRI como teste índice, um resultado pré-especificado de nossa pesquisa, a metanálise foi inviável devido à disponibilidade limitada de dados. Apenas dois estudos forneceram dados específicos do SPERRI,^26,27^ enquanto os outros combinaram o SPERRI com o APERP, dificultando o isolamento de dados especificamente relacionados apenas ao SPERRI.

**Figura 4:**
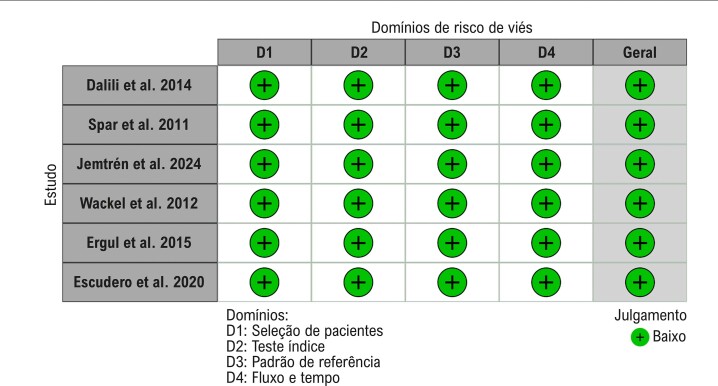
– Risco de viés dos estudos incluídos de acordo com a ferramenta QUADAS-2.

Em nossa análise de risco de viés usando a ferramenta QUADAS-2, identificamos que todos os estudos apresentam metodologia satisfatória com baixo risco de viés. (Figura 4).

## Discussão

Durante nossa revisão sistemática e metanálise, encontramos um aspecto de variabilidade entre os estudos que impactou nossa interpretação: as diferentes definições do que constitui um teste positivo verdadeiro. Esse problema provavelmente surge porque a hipótese nula, ou suposição básica, inicialmente postula a presença de uma via acessória, com a mudança — ou rejeição dessa hipótese nula — sendo a perda da pré-excitação ventricular. Paradoxalmente, esse resultado indica um risco menor, o que levou a um padrão na literatura caracterizado por baixa sensibilidade e alta especificidade.

Embora não seja necessariamente incorreto que alguns estudos tenham definido indivíduos “doentes” como aqueles de baixo risco (em vez de alto risco), isso criou um problema de inconsistência na literatura. Por exemplo, no estudo de Sharma et al.,^30^ que não foi incluído nas fases finais da nossa revisão devido à comparação do teste índice com a morte súbita como teste de referência, as sensibilidades excederam 80% nas análises. Escudero, por exemplo, também definiu os verdadeiros positivos como aqueles que perderam a pré-excitação e apresentaram menor risco, mas interpretou os valores preditivos com mais precisão, afirmando que “o valor preditivo positivo para excluir APs de alto risco foi de 93%”.^28^

Portanto, diante da incerteza sobre se o teste apresenta alta sensibilidade ou especificidade, parece que ele tem sido interpretado incorretamente por um longo tempo. Se considerado um teste de baixa sensibilidade, como se pensava anteriormente, muitos poderiam interpretá-lo como uma falha em descartar vias de alto risco. Contudo, esse não é o caso. Como definimos cuidadosamente indivíduos “doentes” como aqueles com vias de alto risco, e um teste positivo como aquele em que a via acessória não desaparece durante o teste ergométrico, um teste altamente sensível é, por definição, capaz de descartar vias de alto risco. O valor preditivo negativo, que é um cálculo dependente da prevalência da doença em estudos,^31^ acaba sendo alto.

Uma maneira clinicamente mais esclarecedora de interpretar os resultados é considerar as razões de verossimilhança.^17^ A razão de verossimilhança negativa é de 0,260, o que indica que a presença de uma via acessória de alto risco é aproximadamente quatro vezes menos provável diante de um teste negativo (ou seja, um teste que mostra perda de pré-excitação) em comparação com o que aconteceria se esse resultado não tivesse sido observado.^32^ Embora a redução da probabilidade de uma via de alto risco por um fator de quatro seja certamente um achado relevante, os autores acreditam que essa redução, por si só, não é suficiente para classificar esse teste como uma ferramenta definitiva para estratificar vias acessórias de alto ou baixo risco. Para esse propósito, o estudo eletrofisiológico, que permanece o padrão ouro para avaliar as propriedades de condução anterógrada da via, continua sendo a abordagem mais recomendada pelas diretrizes atuais (Figura Central).

**Figura Central:**
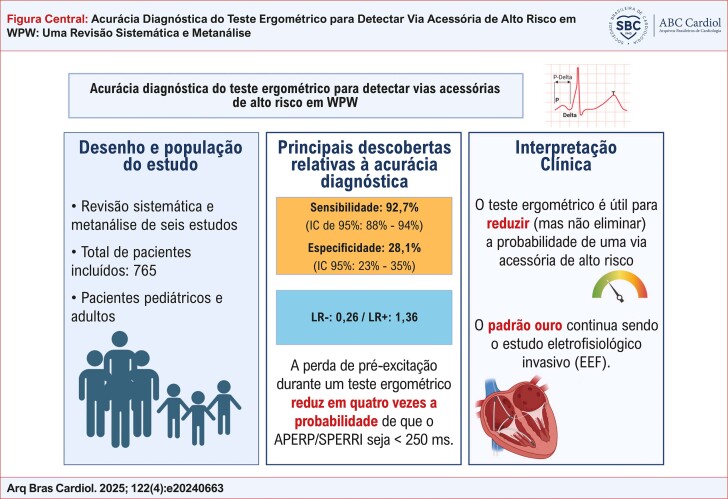
: Acurácia Diagnóstica do Teste Ergométrico para Detectar Via Acessória de Alto Risco em WPW: Uma Revisão Sistemática e Metanálise

A análise de sensibilidade, que excluiu populações adultas e se concentrou apenas em pacientes pediátricos (ou menores de 21 anos), demonstrou que o desempenho do teste é consistente. Esse achado ressalta a robustez da nossa análise ao mostrar resultados semelhantes em diferentes populações.

Esta metanálise produziu resultados esclarecedores ao consolidar descobertas de estudos anteriores em dados agrupados, estabelecendo um precedente para padronizar definições em pesquisas futuras para evitar confusão e conclusões incorretas,^13^ como a noção de que “a perda de uma pré-excitação não tem o poder de reduzir a probabilidade de uma via acessória de alto risco”. A padronização é vital para unificar diversos estudos sobre a síndrome de WPW, garantindo uma interpretação consistente de testes não invasivos.

### Limitações

Embora esta revisão sistemática e metanálise ofereçam insights abrangentes, diversas limitações devem ser observadas. Um desafio primário surgiu da inconsistência na forma como os estudos definiram resultados “verdadeiros positivos”, resultando em variações significativas na sensibilidade e especificidade relatadas. Essa discrepância decorre de diferentes interpretações e aplicações de critérios diagnósticos entre os estudos, o que pode influenciar os resultados da nossa metanálise. Reconhecemos que a nossa redefinição de quem é considerado “doente” ou “saudável” com base nos resultados dos testes pode parecer contraintuitiva. No entanto, optamos por manter essa abordagem porque ela impacta significativamente a orientação da curva SROC. Usar as definições tradicionais predominantes na literatura teria produzido uma curva oposta. Acreditamos que essa abordagem fornece uma compreensão mais clara da utilidade diagnóstica do teste na identificação de vias de alto risco, embora possa desafiar interpretações convencionais.

A generalização dos nossos resultados pode ser prejudicada pelo número limitado de estudos que atendem aos nossos critérios de inclusão. Com apenas seis estudos incluídos, e apenas dois fornecendo dados específicos sobre SPERRI, nossa capacidade de tirar conclusões amplas, especialmente em relação ao SPERRI, é de certa forma restrita. Além disso, ao considerar populações pediátricas, a presença de anomalias cardíacas congênitas, como a Anomalia de Ebstein, não foi avaliada separadamente. Reunir todos os resultados pediátricos para fornecer uma conclusão geral sobre a sensibilidade e especificidade do teste pode não apresentar precisão, pois a presença dessas anomalias pode alterar distintamente o desempenho diagnóstico do teste.

Além disso, em cenários práticos, um problema notável é a variabilidade inter e intraobservador, decorrente da dificuldade de observar a perda de pré-excitação em um ECG frequentemente repleto de artefatos de movimento durante o teste ergométrico. Entretanto, nenhum dos estudos incluídos avaliou esse resultado e, portanto, nossa metanálise não pôde abordar essa questão.

A heterogeneidade nos desenhos dos estudos e nas características dos participantes também representa um desafio. Variações nos cenários e nos perfis dos participantes entre os estudos incluídos podem limitar a aplicabilidade de nossas descobertas a populações mais amplas de WPW.

Por fim, confiar em dados publicados, sem acesso a dados individuais dos pacientes, limita a profundidade da nossa análise. Apesar das tentativas de obter informações adicionais dos autores, a falta de respostas prejudicou nossa capacidade de realizar análises de subgrupos mais detalhadas e confirmar a robustez dos achados em diferentes subgrupos de pacientes.

## Conclusão

Nossa revisão sistemática e metanálise sintetizaram efetivamente as evidências disponíveis sobre a acurácia diagnóstica de testes ergométricos não invasivos para detectar vias acessórias de alto risco em pacientes com síndrome de Wolff-Parkinson-White.

No entanto, é importante observar que, embora as descobertas sugiram que a perda repentina de pré-excitação reduza a probabilidade de uma via de alto risco, isso não significa necessariamente descartar completamente as condições de alto risco. A redução na probabilidade em aproximadamente quatro vezes indica uma utilidade diagnóstica razoável, mas não definitiva. Os médicos devem interpretar esses resultados com cautela e considerá-los como parte de uma estratégia diagnóstica mais ampla, incorporando outros fatores clínicos e ferramentas de diagnóstico para garantir uma avaliação de risco abrangente em pacientes com síndrome de WPW.
