# Supplementary material for: Acurácia Diagnóstica do Teste Ergométrico para Detectar Via Acessória de Alto Risco em WPW: Uma Revisão Sistemática e Metanálise
Source: Arq Bras Cardiol. 2025 Apr 17;122(4):e20240663. [Article in Portuguese] doi: 10.36660/abc.20240663 (PMC12107794; doi:10.36660/abc.20240663)
Supplement: Translation

# Translation: Diagnostic Test Accuracy of Exercise Testing in Detecting High-Risk Accessory Pathways in WPW: A Systematic Review and Meta-Analysis

**DOI:** 10.36660/abc.20240663i

**Authors:** José Nunes de Alencar, Fabio Mahamad Rassi, Raquel Pereira Rios, Matheus Kiszka Scheffer, Guilherme Dagostin de Carvalho

**Affiliations:** 1 Brazil Instituto Dante Pazzanese de Cardiologia, São Paulo, SP – Brazil; 2 Brazil Escola Paulista de Medicina da Universidade Federal de São Paulo, São Paulo, SP – Brazil

**Keywords:** Wolff-Parkinson-White Syndrome, Exercise Test, Systematic Review, Meta-Analysis

## Abstract

**Background:**

Wolff-Parkinson-White (WPW) syndrome is characterized by ventricular pre-excitation, which can lead to severe arrhythmic events such as supraventricular tachycardia and pre-excited atrial fibrillation. The diagnostic value of non-invasive exercise tests in detecting high-risk accessory pathways remains inconsistent in the literature.

**Objectives:**

To evaluate the diagnostic accuracy of non-invasive exercise tests compared to invasive electrophysiological studies (EPS) for identifying high-risk accessory pathways in WPW syndrome.

**Methods:**

Following PRISMA-DTA guidelines, a comprehensive search was conducted in PubMed, Scopus, and Web of Science databases. Eligible studies assessed the sensitivity, specificity, and likelihood ratios of non-invasive exercise tests in WPW patients, using EPS as the reference standard. A bivariate random-effects model was applied for meta-analysis.

**Results:**

Six studies, comprising a total of 765 patients, met the inclusion criteria. The pooled sensitivity was 92.7% (95% CI: 88.0% – 94.0%), while the pooled specificity was 28.1% (95% CI: 23% – 35.1%). A negative likelihood ratio (LR-) of 0.260 (95% CI: 0.174 – 0.387) indicated that the presence of a high-risk accessory pathway is about four times less likely after a negative test result. Sensitivity analysis restricted to pediatric patients showed consistent results.

**Conclusion:**

Non-invasive exercise tests demonstrate a reasonable diagnostic utility for ruling out high-risk pathways in WPW syndrome. However, caution is advised when using these tests as standalone criteria for risk stratification.

## Introduction

Ventricular pre-excitation, a condition affecting around 0.1% of neonates,^1^ can manifest clinically throughout life with symptoms ranging from palpitations and syncope to more severe outcomes, including sudden cardiac death. This is largely due to its association with supraventricular tachycardia and atrial fibrillation. Patients diagnosed with Wolff-Parkinson-White (WPW) syndrome face a notably higher mortality rate, with reported sudden death incidents occurring at approximately 0.15% annually, potentially escalating to 3–4% over a lifetime.^2^

Clinical and electrophysiological characteristics associated with an increased risk of sudden cardiac death in WPW syndrome hinge on the accessory pathway’s ability for rapid atrioventricular conduction. Key indicators of heightened risk include a shortest pre-excited RR interval (SPERRI) < 250 ms or a notably short antegrade effective refractory period of the accessory pathway (APERP), ranging between 220–270 ms.^3^ Furthermore, the abrupt and complete normalization of the PR interval, along with the disappearance of the delta wave during exercise testing, has traditionally been recognized as a low-risk marker.^8,9^ Non-invasive evaluation of the conducting properties of the accessory pathway may be considered (Class IIb) in individuals with asymptomatic pre-excitation, according to ESC guidelines.^7^

This systematic review and meta-analysis follows PRISMA-DTA guidelines^10^ and aims to synthesize and analyze evidence across studies to evaluate the sensitivity, specificity, likelihood ratios, and diagnostic odds ratios of exercise tests in this context.

## Methods

The protocol for this systematic review and meta-analysis of diagnostic test accuracy (DTA) has been registered with the International Prospective Register of Systematic Reviews (PROSPERO). The registration number for accessing the protocol is CRD42024526932.

We conducted a thorough research in the PubMed, Scopus, and Web of Science databases, with the final search conducted on 3/20/24. The search strategy was designed to encompass terms related to WPW syndrome, non-invasive exercise testing, and diagnostic outcomes. For PubMed, terms such as “Wolff-Parkinson-White Syndrome,” “preexcitation,” “exercise test,” “APERP,” “SPERRI,” and related keywords were included. Similar search strategies were adapted for Scopus and Web of Science, considering the syntax and search capabilities of each database.

Studies eligible for inclusion were those that assessed the diagnostic accuracy of non-invasive exercise tests in detecting high-risk accessory pathways in Wolff-Parkinson-White (WPW) syndrome patients, with invasive electrophysiological studies (EPS) serving as the reference standard. Participants of any age diagnosed with WPW syndrome, who underwent both non-invasive exercise testing and invasive EPS, were considered. The primary outcomes examined included sensitivity, specificity, positive likelihood ratio, and negative likelihood ratio of exercise tests in predicting the risk of arrhythmia. We included observational studies, retrospective analyses, and prospective cohort studies published in any language from inception to the present. Exclusion criteria comprised reviews, case reports, and studies lacking clear diagnostic outcome measures or a direct comparison between the index test and the reference standard.

The title screening phase of our systematic review was conducted by two independent researchers (RR and FR) using the HubMeta platform.^11^ Any discrepancies identified during the initial screening were resolved by a third independent researcher (MS). Full-text screening was then carried out by another pair of independent researchers (JA and GD). In cases of disagreements, the issues were resolved through discussion among the authors to reach a consensus.

During the data extraction phase of our systematic review, we encountered a recurring inconsistency in the literature regarding the definitions of what constitutes a positive test and how “disease” status is determined. This inconsistency affected the classification of true and false positives, as well as true and false negatives.^12^ Commonly, studies consider a test positive if there is a sudden loss of ventricular pre-excitation on the ECG during exercise, thereby labeling individuals as “low risk,” identified by an APERP/SPERRI > 250 ms. In our approach, we classify individuals confirmed to be at low risk (APERP/SPERRI > 250 ms) and who lose pre-excitation as “true negatives,” meaning they are “truly absent of risk.” Consequently, we defined a positive test as one where pre-excitation is not lost, and a “truly diseased” individual (“true positive”) is defined as one at high risk, identified by an APERP/SPERRI ≤ 250 ms.

This adjustment means that what we measure as sensitivity in our study corresponds to what the original authors might have reported as specificity. Similarly, the positive predictive value (PPV) and negative predictive value (NPV) have been swapped. This decision, though challenging, was pivotal, as we believe it will yield more robust results and facilitate a clearer understanding among the medical community regarding risk stratification in WPW syndrome.

To evaluate bias risk and applicability concerns within each study, we utilized the QUADAS-2 tool.^13^ This thorough assessment addressed various aspects, including patient selection, the index test, the reference standard, and flow/timing. Additionally, we employed the robvis visual tool to display bias risk assessments across studies.^14^

### Statistical analysis

Studies data were organized into an Excel spreadsheet that captures essential metrics, such as true positives, false positives, true negatives, and false negatives. To guarantee the accuracy and completeness of the extracted information, efforts were made to contact the authors of the studies for any clarifications or additional data. Following this, a bivariate random-effects model was employed to pool sensitivity and specificity estimates across studies.^15,16^ This approach accounts for the potential heterogeneity and correlation between sensitivity and specificity within each study. The model also calculated related findings, including likelihood ratios and the Diagnostic Odds Ratio (DOR). The positive likelihood ratio (LR+) quantifies how much the probability of disease increases with a positive test result. In contrast, the negative likelihood ratio (LR-) reflects how much the probability of disease decreases with a negative test result. These metrics are considered more applicable to clinical practice than sensitivity and specificity because they incorporate a probabilistic reasoning framework.^17^ The DOR can be interpreted as the ratio of the odds of disease in test positives relative to the odds of disease in test negatives, providing a single measure of test effectiveness.^18^

The analyses were facilitated by the MetaDTA software (version 2.0.5),^19,20^ which is specifically designed for diagnostic test accuracy meta-analyses. Forest plots were used to visually represent the sensitivity and specificity distributions across studies and their pooled estimates.

To quantify statistical heterogeneity, we used the Bayesian I^2^ statistic^21,22^ and the area of the 95% prediction ellipse.^23^

## Results

Our systematic review and meta-analysis included six studies,^24-29^ encompassing a total of 765 patients (Figure 1). The details of these studies are summarized in Table 1.

**Figure 1:** – PRISMA Flowchart of Study Selection for Systematic Review and Meta-Analysis.

**Table 1:** – Studies data

Study	Population	Sample size	Index test	Number of tested patients	Reference test
Dalili et al.^26^ 2014	Pediatric patients	37	Loss of pre-excitation in exercise test	27	SPERRI and APERP < 250 ms
Spar et al.,^24^ 2011	Age < 21y	76	Sudden Loss of pre-excitation in exercise test	76	APERP < 270 ms
Jemtrén et al.,^29^ 2024	Average age of 39y, symptomatic and asymptomatic patients	164	Sudden Loss of pre-excitation in exercise test	164	APERP or SPERRI ≤ 250 ms
Wackel et al.,^25^ 2012	Pediatric patients	135	Low risk in any non-invasive test	76	APERP or SPERRI ≤ 250 ms
Ergul et al.,^27^ 2015	Pediatric patients	40	Sudden Loss of pre-excitation in exercise test	40	SPERRI and APERP < 250 ms
Escudero et al.,^28^ 2020	Age < 21y	1589	Sudden Loss of pre-excitation in exercise test	382	SPERRI and APERP < 250 ms

Baseline data of individual studies.

Regarding APERP or SPERRI, the pooled sensitivity, which measures the ability of the test to detect true positives (those who do not lose ventricular pre-excitation during the exercise stress test) among high-risk individuals (those with APERP/SPERRI ≤ 250 ms), was 92.7%. The pooled specificity, indicating the test’s ability to identify true negatives (those who lose ventricular pre-excitation) among low-risk individuals, was 28.1%. Figure 2 summarizes point estimates and 95% confidence intervals. The positive likelihood ratio (LR+) was 1.29 (95% CI: 1.179 – 1.411), and the negative likelihood ratio (LR-) was 0.260 (95% CI: 0.174 – 0.387). The DOR was 4.962 (95% CI: 3.122 – 7.885).

**Figure 2:** – Forest plots representing the sensitivity and 1-specificity (false positive rates) of each included study in detecting the sudden loss of pre-excitation during exercise testing as a marker for low-risk accessory pathways. Each point estimates the sensitivity and 1-specificity for an individual study, accompanied by confidence intervals (CIs). The bottom line displays the prediction interval, indicating the expected range of sensitivities if the test were applied in different settings. The pooled estimates are discussed in the main text.

In terms of heterogeneity analysis, we observed a Bayesian I^2^ index of 29% for sensitivity and 77% for specificity. The area of the ellipse in the Summary Receiver Operating Characteristic (SROC) curve was 0.046, indicating a low level of heterogeneity (Figure 3).

**Figure 3:** – Summary Receiver Operating Characteristic (SROC) curve displaying the trade-off between sensitivity and false positive rate for predicting high-risk accessory pathways. The SROC curves summarize overall diagnostic accuracy.

In the sensitivity analysis, it was pertinent to exclude the study by Jemtrén et al.,^29^ which uniquely included adults over the age of 21. This sensitivity analysis aimed to evaluate the accuracy of the study in the pediatric population, and the results were as follows: The pooled sensitivity was 92.3% (95% CI: 88.8% – 94.8%), and the pooled specificity was 28.4% (95% CI: 21.3% – 36.8%). The positive likelihood ratio (LR+) was 1.29 (95% CI: 1.161 – 1.433), and the negative likelihood ratio (LR-) was 0.270 (95% CI: 0.179 – 0.408).

Regarding the use of SPERRI as the index test, a pre-specified outcome of our research, meta-analysis was unfeasible due to limited data availability. Only two studies provided specific SPERRI data,^26,27^ while other studies combined SPERRI with APERP, hindering the isolation of data specifically related to SPERRI alone.

In our risk of bias analysis using the QUADAS-2 tool, we identified that all studies exhibit satisfactory methodology with a low risk of bias. (Figure 4).

**Figure 4:** – Risk of bias of the included studies according to the QUADAS-2 tool.

## Discussion

During our systematic review and meta-analysis, we encountered an aspect of variability across studies that impacted our interpretation: the differing definitions of what constitutes a true positive test. This issue likely arises because the null hypothesis, or the baseline assumption, initially posits the presence of an accessory pathway, with the change—or rejection of this null hypothesis—being the loss of ventricular pre-excitation. Paradoxically, however, this result indicates a lower risk. This has led to a pattern in the literature characterized by low sensitivity and high specificity.

While it is not necessarily erroneous that some studies have defined “diseased” individuals as those at low risk (rather than high risk), this has created a problem of inconsistency across the literature. For example, in the study by Sharma et al.,^30^ which was not included in the final phases of our review due to its comparison of the index test with sudden death as the reference test, sensitivities exceeded 80% in their analyses. Escudero, for instance, also defined true positives as those who lost pre-excitation and had lower risk but interpreted predictive values more accurately, stating that “the positive predictive value for excluding high-risk APs was 93%”.^28^

Therefore, in the face of the uncertainty of whether the test exhibits high sensitivity or specificity, it seems there has been a longstanding misinterpretation of the test. If it is considered a low-sensitivity test, as previously thought, many might interpret this to mean that it fails to rule out high-risk pathways. However, this is not the case. As we have carefully defined “diseased” individuals as those with high-risk pathways and a positive test as one where the accessory pathway does not disappear during exercise testing, a highly sensitive test is, by definition, capable of ruling out high-risk pathways. The negative predictive value, which is a calculation dependent on the disease’s prevalence in studies,^31^ ends up being high.

A more clinically insightful way to interpret the results is to consider the likelihood ratios.^17^ The negative likelihood ratio is 0.260, implying that the presence of a high-risk accessory pathway is approximately four times less likely in the face of a negative test (i.e., a test that shows loss of pre-excitation) compared to if this result had not been observed.^32^ While reducing the likelihood of a high-risk pathway by a factor of four is certainly a relevant finding, the authors believe this reduction alone is not sufficient to establish this test as a definitive tool for stratifying high- or low-risk accessory pathways. For this purpose, the electrophysiological study, which remains the gold standard for assessing antegrade conduction properties of the pathway, continues to be the most recommended approach by current guidelines (Central illustration).

**Central Illustration:** : Diagnostic Test Accuracy of Exercise Testing in Detecting High-Risk Accessory Pathways in WPW: A Systematic Review and Meta-Analysis

The sensitivity analysis, which excluded adult populations and focused solely on pediatric patients (or those under 21 years of age), demonstrated that the test performance was consistent. This finding underscores the robustness of our analysis by showing similar results across different populations.

This meta-analysis has yielded insightful results by consolidating findings from previous studies into pooled data and setting a precedent for standardizing definitions in future research to prevent confusion and incorrect conclusions,^13^ such as the notion that “the loss of a pre-excitation lacks the power to reduce the probability of a high-risk accessory pathway.” Standardization is vital for unifying diverse studies on WPW syndrome, ensuring a consistent interpretation of non-invasive tests.

### Limitations

While this systematic review and meta-analysis provide comprehensive insights, several limitations should be noted. A primary challenge arose from the inconsistency in how studies defined “true positive” results, leading to significant variations in reported sensitivity and specificity. This discrepancy stems from differing interpretations and applications of diagnostic criteria across studies, which could potentially influence our meta-analysis findings. We acknowledge that our redefinition of who is considered “diseased” or “healthy” based on test outcomes may seem counterintuitive. However, we opted to maintain this approach because it significantly impacts the orientation of the SROC curve. Using the traditional definitions prevalent in the literature would have yielded an opposite curve. We believe this approach offers a clearer understanding of the test’s diagnostic utility in identifying high-risk pathways, though it may challenge conventional interpretations.

The generalizability of our results may be constrained by the limited number of studies meeting our inclusion criteria. With only six studies included and just two providing specific SPERRI data, our ability to draw broad conclusions, especially regarding SPERRI, is somewhat restricted. Moreover, when considering pediatric populations, the presence of congenital cardiac abnormalities, such as Ebstein’s Anomaly, was not evaluated separately. Combining all pediatric results to provide a general conclusion about the sensitivity and specificity of the test may lack precision, as the presence of these anomalies can distinctly alter the test’s diagnostic performance.

Furthermore, in practical settings, a notable issue is inter- and intra-observer variability, stemming from the difficulty of observing the loss of pre-excitation on an ECG often filled with movement artifacts during exercise testing. However, none of the included studies evaluated this outcome, and therefore, our meta-analysis could not address this issue.

The heterogeneity in study designs and participant characteristics also presents a challenge. Variations in settings and participant profiles among the included studies may limit the applicability of our findings to broader WPW populations.

Lastly, the reliance on published data, without access to individual patient data, limits the depth of our analysis. Despite attempts to obtain additional information from authors, the lack of responses hindered our ability to conduct more detailed subgroup analyses and confirm the robustness of findings across different patient subgroups.

## Conclusion

Our systematic review and meta-analysis have effectively synthesized the available evidence on the diagnostic accuracy of non-invasive exercise tests for detecting high-risk accessory pathways in patients with Wolff-Parkinson-White syndrome.

However, it is important to note that while the findings suggest that the sudden loss of pre-excitation reduces the likelihood of a high-risk pathway, this does not necessarily rule out high-risk conditions entirely. The reduction in likelihood by approximately four times indicates reasonable, but not definitive, diagnostic utility. Clinicians should interpret these results with caution, using them as part of a broader diagnostic strategy, incorporating other clinical factors and diagnostic tools to ensure a comprehensive risk assessment for patients with WPW syndrome.
